# Evaluation of HER2-specific peptide ligand for its employment as radiolabeled imaging probe

**DOI:** 10.1038/s41598-018-21283-3

**Published:** 2018-02-14

**Authors:** Hadis Honarvar, Enrica Calce, Nunzianna Doti, Emma Langella, Anna Orlova, Jos Buijs, Valentina D’Amato, Roberto Bianco, Michele Saviano, Vladimir Tolmachev, Stefania De Luca

**Affiliations:** 1 Department of Surgical Sciences, Radiology, Uppsala University Hospital, Uppsala, Sweden; 2 Institute of Biostructures and Bioimaging, National Research Council, Naples, Italy; 3 Division of Molecular Imaging, Department of Medicinal Chemistry, Uppsala University, Uppsala, Sweden; 4 Department of Clinical Medicine and Surgery, University of Naples “Federico II”, Naples, Italy; 5 Institute of Crystallography, National Research Council, Bari, Italy; 6 Department of Immunology, Genetics and Pathology, Uppsala University, Uppsala, Sweden

## Abstract

HER2 transmembrane receptor is an important target in immunotherapy treatment of breast and gastroesophageal cancer. Molecular imaging of HER2 expression may provide essential prognostic and predictive information concerning disseminated cancer and aid in selection of an optimal therapy. Radiolabeled low molecular weight peptide ligands are particularly attractive as probes for molecular imaging, since they reach and bind to the target and clear from non-target organs and blood stream faster than bulky antibodies. In this study, we evaluated a potential HER2-imaging probe, an A9 nonapeptide, derived from the trastuzumab-Fab portion. Its cellular uptake was investigated by mass spectrometry analysis of the cytoplasmic cellular extracts. Moreover, based on *in-silico* modeling, DTPA chelator was conjugated to N-terminus of A9. ^111^In-labeled A9 demonstrated nanomolar affinity to HER2-expressing BT474 cells and favorable biodistribution profile in NMRI mice. This study suggests that the peptide A9 represents a good lead candidate for development of molecular probe, to be used for imaging purposes and for the delivery of cytotoxic agents.

## Introduction

HER2 is a transmembrane protein, belonging to the ErbB or epidermal growth factor receptor (EGFR) family^1^. Structurally it is featured by an N-terminal extracellular ligand binding portion (ECD), a single alpha-helix transmembrane segment (TM), and an intracellular protein tyrosine kinase. Overexpression of HER2 is associated with a wide number of cancers, including lung, breast, and ovarian, as well as adenocarcinomas of colon and salivary gland^2,3^. In particular, HER2 receptor is found to be overexpressed in about 30% of primary breast cancers^4,5^, mainly because of its gene amplification. HER2 has a great tendency to dimerize with other ErbB family receptors, which results in activation of the HER signaling pathways^6^. Moreover, it has been found that among all ErbB receptor dimers, the heterodimers containing HER2 receptors have the highest mitogenic potential^7–15^.

HER2 has been extensively investigated, in order to develop new HER2-specific cancer therapies^16,17^. Several studies have focused on immunotherapy of HER2-positive tumors due to its transmembrane accessibility. Trastuzumab (Herceptin) is a humanized IgG1 monoclonal antibody that recognizes an epitope in the extracellular domain (ECD) of HER2^18,19^. It was approved in 1998 by FDA (Food and Drug Administration) for therapy of metastatic breast cancer in combination with cytotoxic chemotherapy^20^. Several strategies have been used to increase the effectiveness of therapy of HER2-positive tumors. For instance, HER2-specific antibodies, their fragments and affibody molecules have been conjugated to cytotoxic molecules^21–24^.

It is essential to select patients with HER2-expressing metastases for HER2-targeting therapy, as this antigen has sufficient expression level only in a fraction of the tumors. A biopsy of primary breast cancer is not informative in this case as discordance in HER2 expression between primary tumor and metastases might be up to 18%^25^. Therefore, radionuclide molecular imaging using radiolabeled anti-HER2 antibodies was suggested, which is a non-invasive approach for determination of HER2 expression in the disseminated breast cancer^20,26–30^. However, intact antibodies are not considered ideal probes for radionuclide molecular imaging due to their long blood residence time, which results in low tumor/blood (T/B) ratios limiting imaging contrast^31^. Moreover, intact IgGs often have a high liver uptake, mediated by interaction of the Fc portion with hepatocyte receptors, which reduces the imaging sensitivity for detection of liver metastases.

Antibody fragments, such as Fab or F(ab’)_2_ have been considered better candidates for tumor imaging. In fact, their relatively small size results in higher extravasation and tissue penetration rates as well as improved blood clearance. Recently, radiolabeled anti-HER2 Fab fragments^32–34^, and novel recombinant antibody forms (scFv, diabodies, minibodies)^35–37^, as well as HER2-binding affibody^38–40^ have been successfully used for imaging purposes.

In this regard, peptide molecules, characterized by low molecular weight, can represent the most appropriate HER2 targeting tracers, since they exhibit superior ability to diffuse across tissues, improving tumor exposure to the drug. Moreover, the peptides are easily produced and the chemistry for their radiolabeling is easier and more flexible compared with monoclonal antibodies and their fragments.

In this context, we have recently developed and studied several trastuzumab (Fab)-derived peptides capable to bind HER2^41^. In particular, the A9 peptide was found to be particularly interesting, since it showed a dissociation constant in nanomolar range for the receptor model HER2-DIVMP^41–43^. Therefore, it was studied as a promising candidate for development of an HER2-specific imaging probe. In this context, the ability of this peptide to be internalized into the tumor cell by receptor-mediated endocytosis has been investigated, since it is considered a crucial step in the process of *in vivo* receptor targeting with radiolabeled peptides^44–46^.

As main goal, we evaluated site-specific conjugation of DTPA chelator to A9, labelling of DTPA-A9 conjugate with radionuclide ^111^In, evaluation of ^111^In-DTPA-A9 binding to living HER2-expressing cancer cells *in vitro*, its stability *in vitro* and *in vivo* and biodistribution of ^111^In-DTPA-A9 *in vivo*.

## Results and Discussion

### Cellular uptake of A9 peptide

The internalization of radiolabeled peptide ligands, upon receptor binding, represents an important event for *in vivo* tumor targeting, since it results in accumulation of radioactive in the tumor tissues. Therefore, it can considerably improve the sensitivity of diagnostic procedures, as well as the efficacy of receptor-mediated radiotherapy.

To this aim, the cytoplasmic localization of A9 peptide was investigated by mass spectrometry analysis in HER2-positive human breast cancer BT474 cells. In particular, A9 peptide was bound to a biotin moiety at its N-terminus, in order to be easily extracted from cytoplasmic cell lysate by using streptavidin-coated magnetic beads. Then, the mixture obtained was analyzed by liquid chromatography-tandem mass spectrometry (LC-MS/MS).

**Figure 1:**
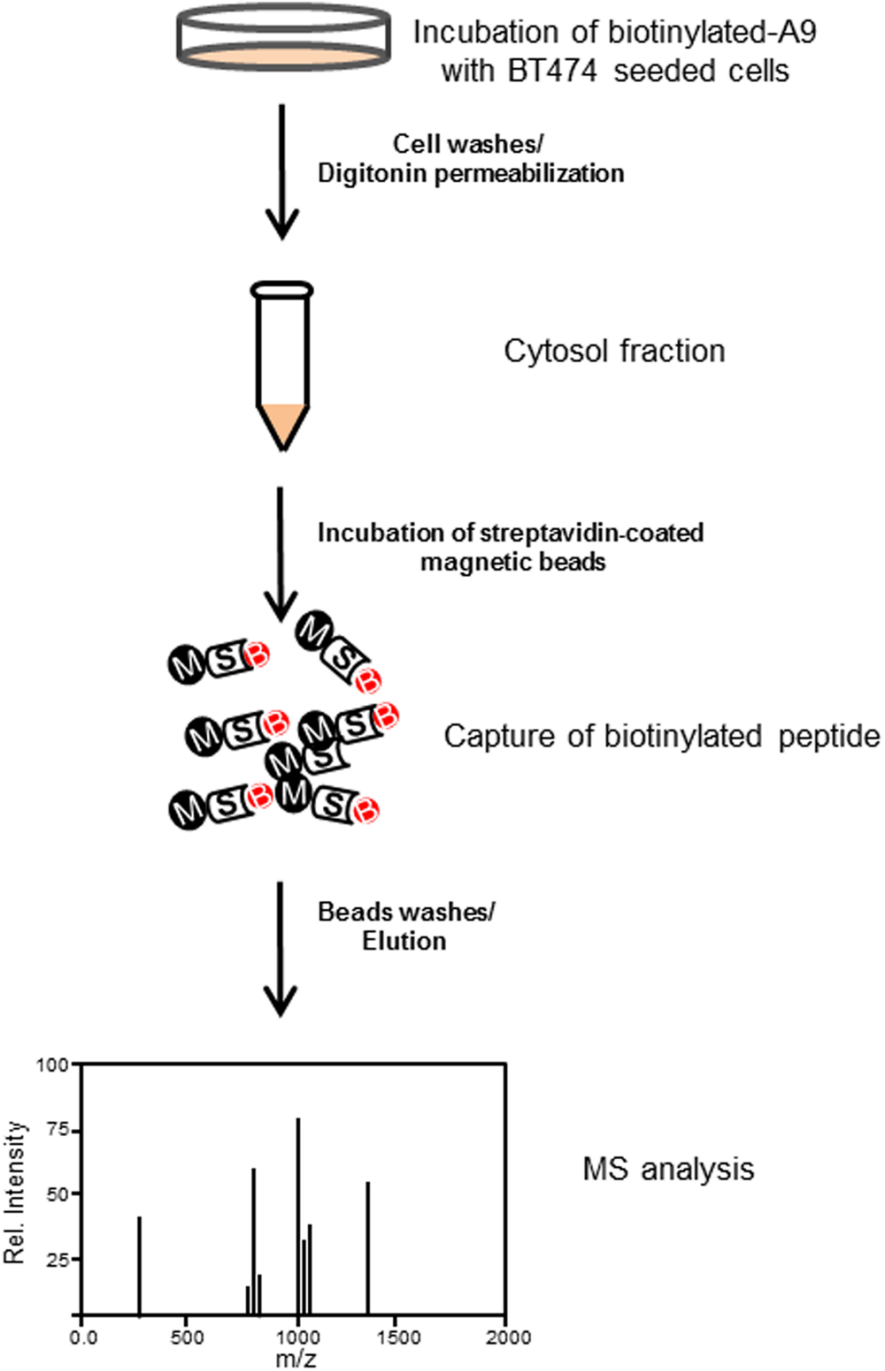
Schematic diagram of affinity tag in an *in vitro* pull-down assay and mass spectrometry analysis.

As shown in Fig. 1 (schematic view of the employed protocol), BT474 cells were treated with biotinylated-A9 for 3 hours, then washed with a PBS buffer in presence of 0.5% Trypsin-Ethylenediaminetetraacetic acid, in order to be collected. The obtained cell pellets were incubated with a digitonin solution to permeabilize the plasma membranes and recover the intracellular fractions^47^. Subsequently, a purification step was performed by incubating the soluble intracellular fraction with the streptavidin-coated magnetic beads, which strongly catch any biotinylated compounds. After filtration, the unbound proteins were removed and the beads were washed with a PBS buffer, before the treatment with a solution of EDTA in formamide at high temperature, which allowed the elution of biotinylated species. The final step consisted of a LC-MS/MS analysis of the collected biotinylated molecules (see Methods section for details). The same procedure was carried out on untreated BT474 cells.

The collected samples were then characterized by using the simple, sensitive and efficient analytical technique LC-MS/MS, in multiple-reaction monitoring (MRM) mode. The MRM strategy provides a unique fragment ion that can be selectively monitored, also in a complex mixture^48^. In order to investigate the fragmentation pattern for biotinylated-A9, pilot LC-MS/MS-MRM experiments were also performed on the synthetic peptide. They allowed us to identify the diagnostic fragment, which is essential for developing a high-sensitivity MRM assay. Therefore, the selection of the most intense ion, among all individual fragment ions, could be performed (see Supplementary Information Fig. S1). In particular, the fragment ion 477.1 m/z was selected as a diagnostic one of the precursor 615.3. On the basis of these data, SRM (selected transition monitoring) mode experiments at m/z 615.0–477.2 on our samples were also performed.

**Figure 2:**
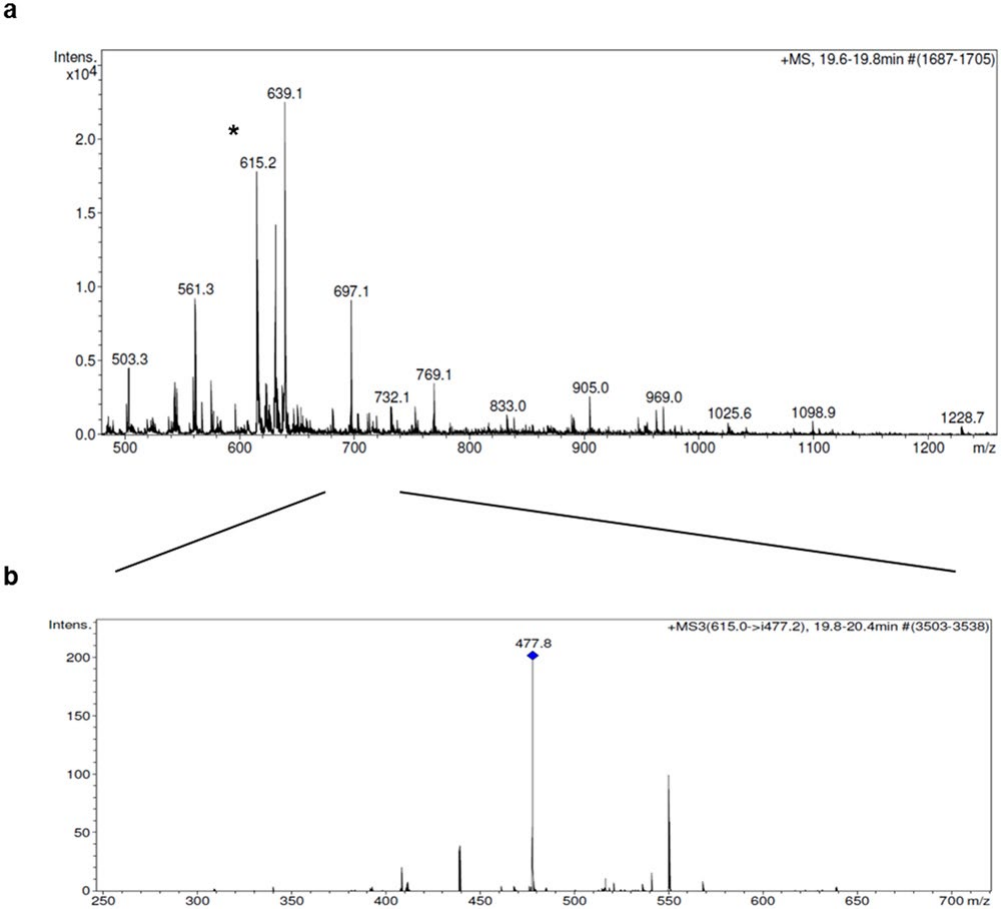
(**a**) MS/MS spectra of the [M + 2 H]^2+^ precursor of biotinylated A9 at 615.2 m/z (*). (**b**) SRM plot of the selected transition at m/z 615.0–477.2.

In Fig. 2a is shown the MS/MS profiles of the samples obtained for BT474 cells incubated with the biotinylated A9, in which the SRM peak is easily detectable (Fig. 2b; see also Supplementary Information Fig. S2). On the other hand, the same SRM peak was not detected in sample collected for untreated BT474 cells, which were used as a negative control (see Supplementary Information Fig. S3).

All these results suggest a putative internalization of A9 peptide inside the cells.

### Design and synthesis of the conjugate A9-DTPA

The final goal of our studies is to develop new targeting agents, which are smaller than antibodies and affibody molecules, and possess a high specificity and affinity for HER2 receptor. They could be used in the non-invasive imaging of HER2-expression level in metastases, thus allowing stratification of patients who would most likely respond to targeted anti-HER2 therapies. This should prevent over- and under treatment and improve efficacy of breast cancer therapy. Therefore, we evaluated the possibility to develop a peptide A9-derivative bearing a chelating agent able to coordinate radioactive metals for applications in cancer diagnosis by nuclear medicine techniques.

**Figure 3:**
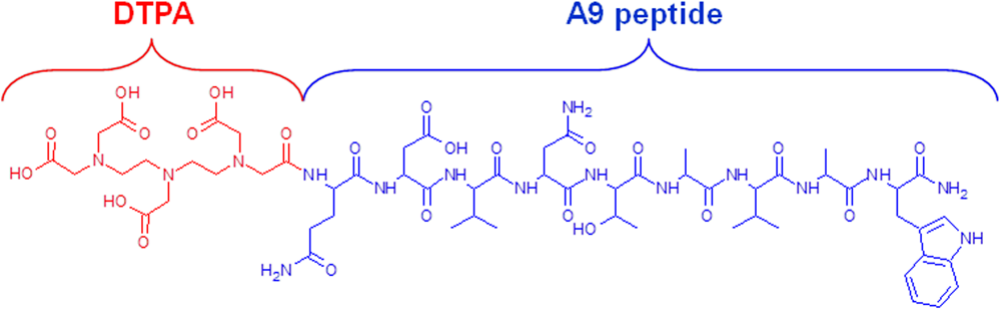
Chemical structure of DTPA-A9 conjugate.

We synthesized the A9 amidated at the C-terminus and coupled with an acyclic chelator at the N-terminus (Fig. 3). These peptide modifications represent a good strategy for stabilizing the peptide molecule and protect it against the exopeptidases. The acyclic chelator DTPA is routinely used to provide *in vitro* and *in vivo* stable complexes of the radioactive isotope ^111^In. The issue of the metal complex stability is crucial for the radiolabeling of peptide molecule: even though many of the chelating agents are both thermodynamically stable and kinetically inert, a complexation of a metal by a chelator is always a reversible process. In addition, some blood proteins, e.g. transferrin, possess metal-complexing properties, which lead to transchelation of a radionuclide from an imaging agent to these proteins^49^. This results in longer retention of a radionuclide in circulation and increases background during imaging. On the other hand, the required stability depends on the residence time of a radiolabeled probe in the circulation. For short peptides, which clear rapidly from blood via kidneys, the exposure to transferrin is short and no measurable transchelation takes place. For example, the heptadentate DTPA provides an adequate *in vivo* stability of binding of ^111^In to octapeptide octreotide, which has a rapid blood clearance^50^, and ^111^In-DTPA-octreotide (OctreoScan) is routinely used in clinics for imaging of neuroendocrine tumors^51^. Taking all these aspects in consideration, we synthesized the conjugate reported in Fig. 3.

**Figure 4:**
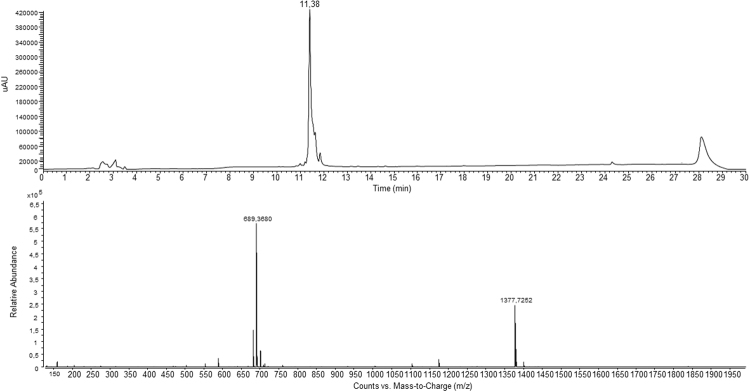
LC-MS profile of the purified DTPA-A9 conjugate.

In more details, the peptide synthesis was performed manually in solid phase using the standard Fmoc-protocol. Rink Amide resin (Merck Millipore) was employed as solid support to release the peptide with the C-terminal amide. The heptadentate DTPA chelator was introduced by using its free carboxylic function to the peptide N-terminus. The final compound was obtained in good yield (60%) and with high purity grade (>98%) after RP-HPLC purification and it was fully characterized for its identity by mass spectrometry (Fig. 4).

### Molecular modeling study

In a recent work, we have studied the putative complex between A9 ligand and the receptor model HER2-DIVMP by employing a computational approach; the identified binding site was further validated by mutagenesis, as well as fluorescence studies^52^.

**Figure 5:**
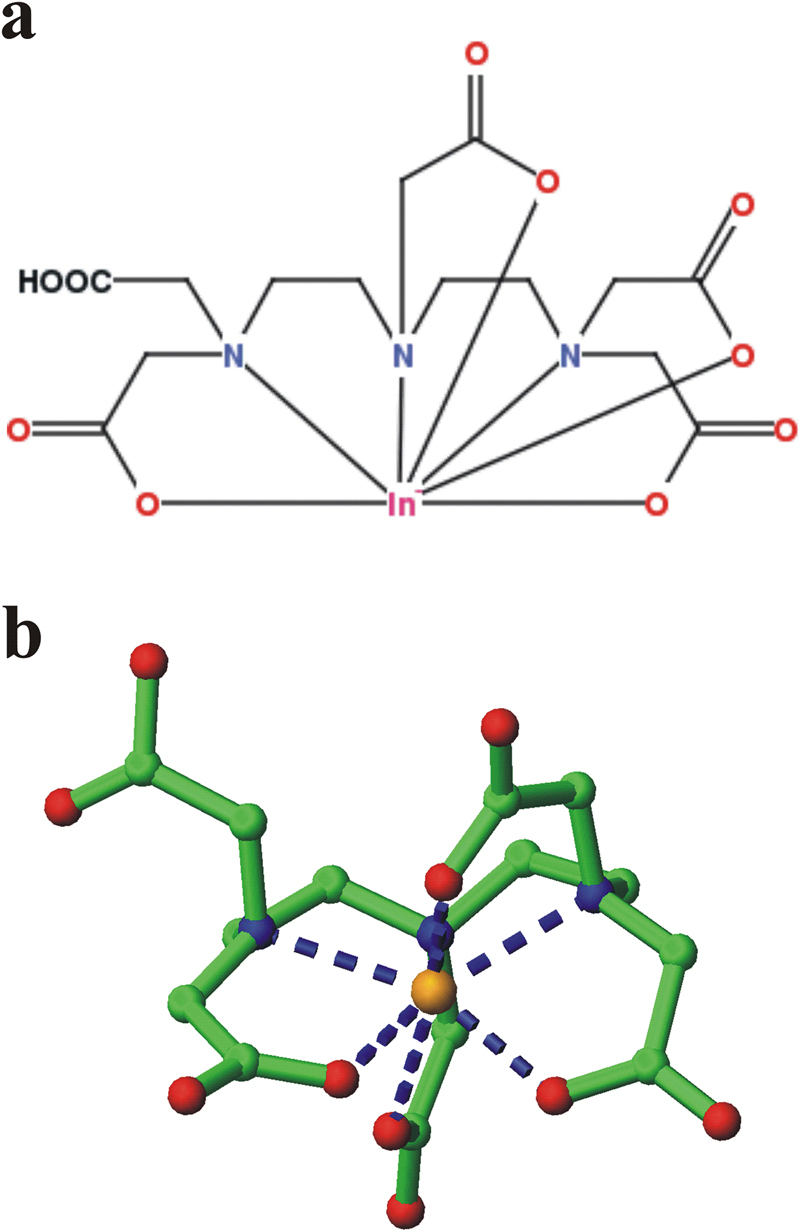
2D diagram (**a**) and 3D image (**b**) of ^111^In-DTPA complex from Cambridge Structural Database (CSD) (code XIKVES)^54^. (**b**) Atoms are displayed in ball-and-sticks mode. Indium atom is in orange.

To investigate the chelating agent site of linkage, which does not interfere on the peptide-receptor interaction, we have modelled the 3D structure of the complex between HER2-DIVMP and ^111^In-DTPA-A9, starting from the previously performed molecular dynamics (MD) study^52^. In details, the structure of ^111^In-DTPA was retrieved from the CSD (The Cambridge Structural Database)^53^ (Fig. 5), whereas a representative conformation of the complex A9/HER2-DIVMP was obtained by our previous Molecular Dynamics study^52^.

**Figure 6:**
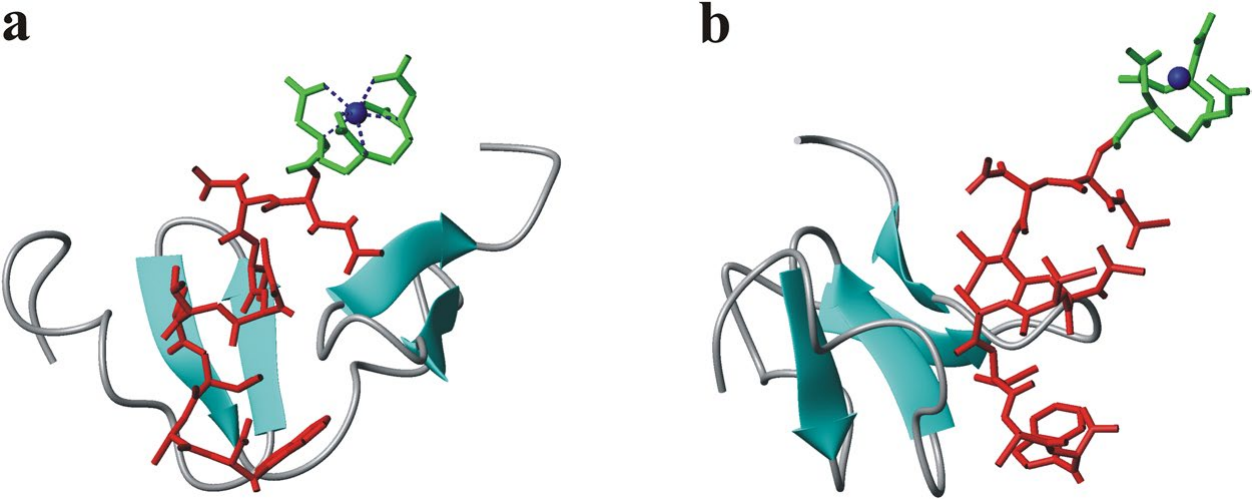
(**a**) Model complex between HER2-DIVMP and ^111^In-DTPA-A9. (**b**) Side-view of the model complex, rotated of 90° along z-axis. HER2-DIVMP receptor is shown in cartoon representation. ^111^In-DTPA-A9 is displayed in sticks (A9: red sticks, DTPA: green sticks).

Subsequently, the model complex between HER2-DIVMP and ^111^In-DTPA-A9 was built through modelling and energy minimization using Insight II package (see Methods section)^54^, it is shown in Fig. 6. The model indicates that the presence of ^111^In-DTPA is compatible with the structure suggested by our previous MD study^52^, since it is directly bounded to the N-terminus of A9, which is not involved into interaction with HER2-DIVMP receptor. A scrambled variant of A9 (SA9) has been designed and synthesized for specificity test. The sequence of scrambled-A9 (WAVQNTDAV) was selected by using a free of charge software.

### Radiolabeling of ^111^In-DTPA-A9

**Figure 7:**
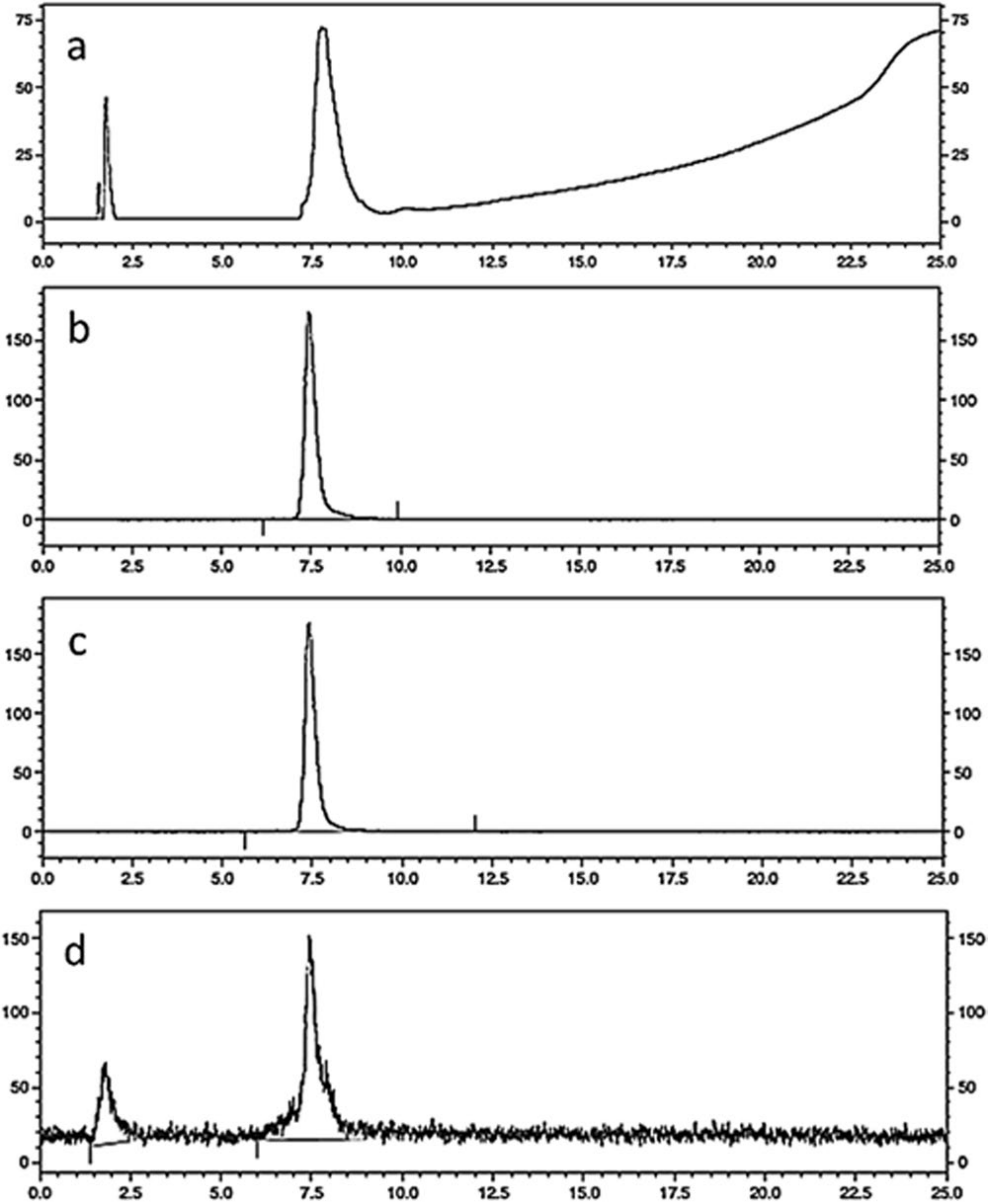
HPLC UV (**a**) and radio-chromatograms (**b**) of a freshly labeled ^111^In-DTPA-A9. (**c**) Radio-chromatogram of murine blood plasma after 60 min incubation with ^111^In-DTPA-A9 (**d**). Radio-chromatogram of murine blood 15 min after injection ^111^In-DTPA-A9.

Both DTPA-A9 and scrambled DTPA-SA9, which was used in specificity test, were labelled with ^111^In in 0.2 M ammonium acetate, pH 5.5, at room temperature. A blank experiment (without adding DTPA-A9) was performed to facilitate interpretation of chromatograms. Radio-HPLC chromatograms are presented in Fig. 7.

As expected, non-conjugated ^111^In was eluted at 2.1 min, immediately after dead volume. The retention time of ^111^In-DTPA-A9 was 7.5 min (Fig. 7a,b) and the retention time of ^111^In-DTPA-SA9 was 7.3 min. Labeling was efficient at room temperature. The analytical radiochemical yield of both^111^In-DTPA-A9 and ^111^In-DTPA-SA9 labelling was over 98%. The specific radioactivity of 1 MBq/µg was achieved.

There was no measurable release of radioactivity after 15 or 60 min (Fig. 7c) of ^111^In-DTPA-A9 incubation with murine blood plasma, which demonstrates its stability against blood-borne peptidases.

### *In vitro* evaluation of kinetics of ^111^In-DTPA-A9 binding to HER2-expressing cells

**Figure 8:**
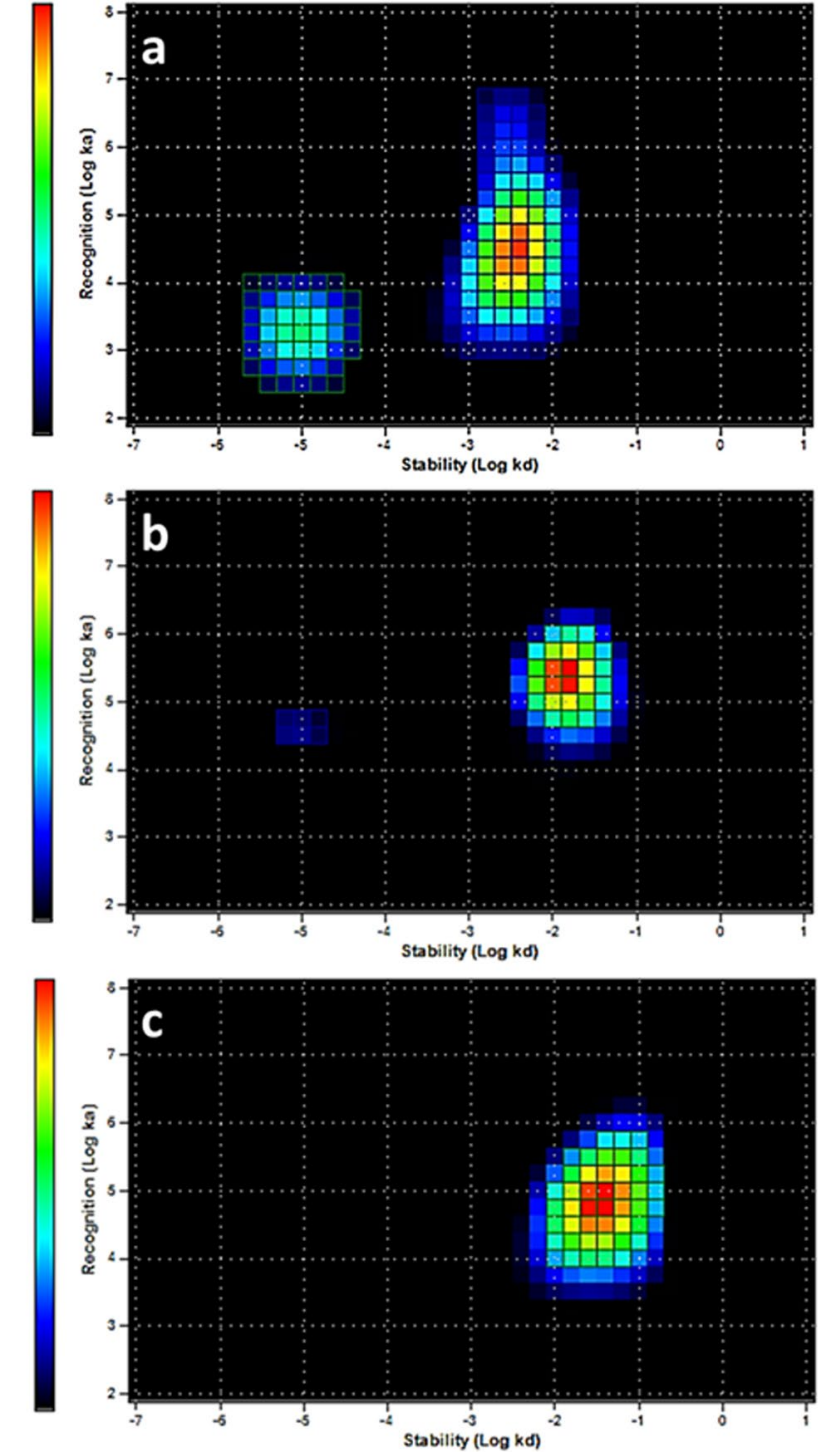
InteractionMaps of ^111^In-DTPA-A9 interaction with HER2-expressing BT474 cells (**a**). Concentrations of ^111^In-DTPA-A9 were 4, 12 and 36 nM. Fitting binding LigandTracer curves to 1:2 interaction models suggest presence of two interactions, one stronger (4.9 nM) and one weaker but predominant (103 nM); (**b**) ^111^In-DTPA-A9 interaction with HER2-expressing BT474 cells in the presence of large excess of trastuzumab. Concentrations of ^111^In-DTPA-A9 and timing were the same as above; (**c**) ^111^In-DTPA-S-A9 interaction with HER2-expressing BT474 cells. Concentrations of ^111^In-DTPA-S-A9 (4, 12 and 36 nM) and timing were the same as for ^111^In-DTPA-A9.

The kinetics of ^111^In-DTPA-A9 binding to and dissociation from HER2-expressing BT474 cells *in vitro* was measured using LigandTracer. The InteractionMap is presented in Fig. 8a. The best fitting of the binding curves was obtained using a 1:2 interaction model. This indicates that the binding of ^111^In-DTPA-A9 to HER2 on living cells is mediated by two binding sites, one strong, with affinity of 4.9 nM, and one weaker, with affinity 103 nM. The presence of two binding sites with different affinities on living cells is intriguing but not unique. Similar phenomenon has been found earlier for binding of ^125^I-labeled anti-HER2 antibody trastuzumab and ^111^In-labeled anti-HER2 affibody molecules to different HER2-expressing cells^55^. One possible explanation for two affinities of ^111^In-DTPA-A9 binding to HER2 might be homo- and heterodimerization of receptors on cancer cells. The dimer formation may slightly change the conformation of receptor, resulting in the change the interaction with a ligand. Similar effect has been demonstrated for other member of HER family, EGFR, by Björkelund and co-workers^56^.

Further elucidation of this phenomenon was obtained by performing LigandTracer experiment in the presence of a large excess of trastuzumab (Fig. 8b). In these conditions, the high affinity interaction was nearly completely suppressed, upon traztuzumab displacement, while low affinity interaction remained. This suggested that this low affinity interaction might be not HER2-specific.

To further clarify this issue, an additional experiment was performed. Specifically, a scrambled conjugate (^111^In-DTPA-SA9), i.e. having the same amino acids of A9 but in a different order, variant of DTPA-A9, was created. As it shown in Fig. 8c, the InteractionMap, evaluating the binding of ^111^In-DTPA-SA9 to Her2-positive BT474 cell, revealed only the low affinity interaction (103 nM).

Taken our results together, the InteractionMap measurements suggest that there are two interactions of ^111^In-DTPA-A9 with HER2-expressing cell. One is HER2-specific, as it can be suppressed by trastuzumab and does not appear in the case of the scrambled variant. Another is non-HER2 specific and might be characteristic for peptides containing the same amino acids as A9. Accordingly, the hypothesis concerning homo- or hetero- dimerization as a cause of low affinity binding should be excluded.

Our previous studies have demonstrated that affinity in a single digit nanomolar range is required for efficient targeting of tumors with high HER2 expression using affibody molecules^57^. A possible way to increase the affinity of A9-based probe to HER2 is a di- or multimerization. Even dimeric form of A9 is small enough to provide both efficient extravasation and rapid renal excretion of an unbound tracer. However, this might require re-modelling and, possibly, designing of a branched linker between N-termini of monomeric units, permitting conjugation of a chelator.

### *In vivo* studies

Specific high affinity binding to molecular target is a necessary precondition for development of an imaging probe, but it is not sufficient. Off-target interaction of the probe with blood and normal tissue might result in undesirable accumulation of an imaging probe in normal organs. Such accumulation would reduce an imaging contrast leading to reduced sensitivity of *in vivo* diagnostics. Particularly, a weak non-HER2-specific interaction found in the InteractionMap experiments might be a reason for undesirable accumulation in normal tissues. Therefore, we measured biodistribution of ^111^In-DTPA-A9 in normal mice at 1 h after injection. Data concerning the biodistribution of ^111^In-DTPA-A9 in BALB/c nu/nu mice 1 h post injection is presented in Table 1. ^111^In-DTPA-A9 showed rapid clearance from blood and normal organs (Table 1). The low (approximately 1% of injected activity) radioactivity in the gastrointestinal tract with content indicates minor role of hepatobiliary excretion in the elimination of the radiolabeled peptide. More apparent was renal excretion with subsequent tubular reabsorption, as kidney uptake of ^111^In-DTPA-A9 was more than 7% ID/g (2% ID/sample).

**Table 1:** Data are presented as average ID%/g and ID%/sample values for 3 animals ± standard deviation. (Data for intestines with content and carcass are presented only as % ID/sample).

organ	% ID/g	% ID/organ (sample)
blood	0.24 ± 0.04	
lung	0.5 ± 0.3	0.06 ± 0.03
liver	0.44 ± 0.04	0.47 ± 0.03
spleen	0.22 ± 0.01	0.02 ± 0.01
stomach	0.27 ± 0.07	0.05 ± 0.06
pancreas	0.13 ± 0.03	0.01 ± 0.00
brain	0.03 ± 0.03	0.00 ± 0.01
kidney	7.0 ± 0.6	2.00 ± 0.1
muscle	0.01 ± 0.01	0.04 ± 0.01
bone	0.16 ± 0.13	0.01 ± 0.01
GI tract (with content)	------------	0.83 ± 0.48
carcass	-------------	3.47 ± 1.01

However, this value was much lower than renal uptake of ^111^In-labeled trastuzumab Fab fragments (53.0 ± 15.94%ID/g at 24 h after injection)^33^, minibodies (16–30%ID/g at 2 h after injection)^34^ or affibody molecules (200–250%ID/g at 4 h after injection)^58^ in murine models. The clearance from blood was rapid, suggesting that the label was stable, and no transchelation to transferrin took place. The radioactivity accumulation was low in liver, lung and bones, i.e. in the major metastatic sites for breast cancer, the main target of anti-HER2 therapies. Thus, the *in vivo* data suggest that ^111^In-DTPA-A9 has favorable biodistribution profile, and the low affinity unspecific interaction does not cause any undesirable accumulation in healthy organs and tissues

It has to be noted that although incubation of A9 *in vitro* with blood serum demonstrated high stability during one hour (see Supplementary Information Fig. S4 and Fig. 7c), peptidases anchored in blood vessels might degrade peptides *in vivo*^59–61^. Therefore, we evaluated stability of ^111^In-DTPA-A9 *in vivo* using the method developed by Nock and co-workers^59^. Our experiment demonstrated that 92% of blood-borne activity was associated with DTPA-A9 at 5 min after injection and ca. 70% at 15 min after injection (Fig. 7d). For comparison, only 5% of activity was bound to mingastrin analogue DOTA-MG11 at 5 min after injection, and the rest was associated with the peptide degradation products. In the case of ^111^In-CP04, over 70% of ^111^In in blood was bound to peptide at 5 min after injection^61^. This was considered as a good stability, and ^111^In-CP04 is currently prepared for clinical study for imaging of medullary thyroid carcinoma. Apparently, ^111^In-DTPA-A9 has sufficient *in vivo* stability.

In conclusion, the ability of A9 peptide to specifically bind the HER2-positive cell plasma membrane with a subsequent internalization was demonstrated. The conjugation of DTPA to N-terminus of A9 via amide bond enables stable labeling with ^111^In. The ^111^In-DTPA-A9 has nanomolar affinity to HER2-expressing cells. The biodistribution data suggest that ^111^In-DTPA-A9 clear rapidly from blood and does not bind to any noticeable extent to any tissues. Thus, ^111^In-DTPA-A9 is a promising starting point for development of HER2-imaging probes and also for optimization of ligands useful for the targeting of HER2-positive tumor tissue.

## Methods

### Materials

Fmoc-protected amino acids, N-hydroxybenzotriazole (HOBT), O-Benzotriazole-N,N,N’,N’-tetramethyl-uroniumhexafluoro- phosphate (HBTU) and Benzotriazolyloxy-tris[pyrrolidino]-phosphonium hexafluorophosphate (PyBOP) were purchased from Calbiochem-Novabiochem (Laufelfingen, Switzerland), piperidine and diisopropylethylamine (DIPEA) were purchased from Fluka (Milwaukee, WI, USA), Rink Amide MBHA resins were purchased from Sigma-Aldrich (St Louis, MO, USA). DTPA(tBu)_4_ was purchased from CheMatech (Dijon, France).

Other solvents were purchased from Sigma-Aldrich or Fluka (Milwaukee, WI, USA) and were used without further purification, unless otherwise stated.

Analytical RP-HPLC runs were carried out on a HP Agilent Series 1100 system using a C18 column, 250*4.6 mm ID (Phenomenex, Torrance, CA, USA) at a flow rate of 1.0 mL min^−1^ or on a C18 monolithic column 50*2 mm ID (Phenomenex, Torrance, CA, USA), operating at 0.6 mL min^−1^. Preparative RP-HPLC was carried out on a Shimadzu 8 A chromatograph coupled with an UV detector, using a C18 column, 22*250 mm (Phenomenex Torrance, CA, USA) at a flow rate of 20 mL min^−1^. For all the RP-HPLC procedures the solvent system used was: H_2_O 0.1% TFA (A) and CH_3_CN 0.1% TFA (B). Separations were achieved applying a linear gradient of B from 20% to 80% in 20 min and monitoring at 210 and 280 nm.

LC–MS data were obtained using a Finnigan Surveyor MSQ single quadrupole or LCQ DECA XP MAX electrospray ionization mass spectrometers, coupled with a Surveyor HPLC (ThermoFisher), operating in the full scan positive mode, between 200 and 2000 m/z.

Buffers, including 0.1 M phosphate buffered saline (PBS), pH 7.5, 0.2 M ammonium acetate, pH 5.5, and 0.2 M citric acid were prepared using common methods from chemicals supplied by Merck (Darmstadt, Germany). High-quality Milli-Q^©^ water (resistance higher than 18 MΩ cm) was used for preparing the solutions. Buffers, which were used for labeling, were purified from metal contamination using Chelex 100 resin (Bio-Rad Laboratories, Richmond, USA). [^111^In]-indium chloride was purchased from Covidien (Hazelwood, US) as a solution in 0.05 M hydrochloric acid. The radiochemical purity of the labelled peptide was analysed using HPLC system from Beckman. Ketalar [ketamine] (50 mg/mL, Pfizer, NY, USA), Rompun [xylazin] (20 mg/mL, Bayer, Leverkusen, Germany), and Heparin (5000 IE/mL, Leo Pharma, Copenhagen, Denmark) were obtained commercially.

The radioactivity was measured using an automated gamma-counter with a 3-inch NaI(Tl) detector (1480 WIZARD, Wallac OY, Turku, Finland). The data were corrected for background.

### Peptide synthesis

The synthesis of A9 and scrambled A9 (SA9) peptides were carried out manually by solid phase method using the standard Fmoc-protecting group strategy. Appropriate Fmoc-amino acid derivatives were employed and a Rink Amide MBHA resin (0.7 mmol g^−1^ substitution; 50 µmol scale) was used as solid support, it releases peptides amidated at C-terminus upon acid treatment. All Fmoc-amino acids were activated by *in situ* PyBop/HOBt//DIPEA activation procedure. Amino acid coupling steps were monitored by Kaiser test after 60 min coupling cycles. Fmoc-deprotection was performed with 20% piperidine in DMF for 5 + 10 min.

After removal of the final N-terminal Fmoc-protecting group, the coupling step with the chelating agent was performed using 2 equiv of DTPA(tBu)_4_ and PyBOP and 4 equiv of DIPEA in DMF. The coupling time was increased to 2 h and completion of the reaction was checked by Kaiser test.

The conjugate cleavage from the solid support and the simultaneous deprotection of all side chains were performed by suspending the fully protected compound-resins in TFA/H_2_O/TIS/EDT/tioanisole (95:2:1:1:1) for 3 h. The peptide conjugate was isolated by precipitation into cold diethyl ether and centrifuged to form a pellet.

The final product was purified by RP-HPLC and analyzed by mass spectrometry.

DTPA-A9, DTPA-SA9: [M + H]^+^ calculated 1377.45 *m/z*, found 1377.7.

For the internalization studies, Biotin was linked to the N-terminus of A9 peptide.

Biotinylated-A9 peptide: [M + H]^+^ calculated 1228.4 *m/z*, found 1228.2. For details see Supplementary Information Figs S5 and S6.

### Internalization studies of A9 in BT474 cells

BT474 cells were seeded at a density of 10^6^ cells/well in 6-well-plates for incubation. After 24 h of preincubation, medium was replaced with fresh medium containing the biotinylated-A9 peptide (at a concentration of 200 μg/mL), and the cells were further incubated for 3 h. The cells were then washed twice with ice cold phosphate buffered saline (PBS; Sigma-Aldrich) before adding ice cold 0.5% Trypsin-Ethylenediaminetetraacetic acid (EDTA; Sigma-Aldrich). Cell pellets and culture media fractions were collected by centrifugation for 10 min at 5,000 rpm and 4 °C. For the subsequent estimation of cellular uptake, BT474 cell pellets were permeabilized with 40 μg/mL digitonin solution (Sigma-Aldrich), which contains 10 mM Hepes, 10 mM KCl, 1.5 mM MgCl_2_, 1 mM DTT, complete protease inhibitors tablet (Roche) at pH 7.4, for 15 min in an ice bath^47^. The supernatants, which represent the intracellular fraction, were collected by centrifugation for 20 min at 16000 rpm and at 4 °C. The Samples obtained were incubated with streptavidin-coated magnetic beads (ThermoFisher Scientific) for 12 h at 4 °C, under continuous agitation, in order to catch the biotinylated-peptide. The streptavidin-bound compounds, after several washes with PBS buffer (pH7.4), were eluted with formamide 95% EDTA 0.5 mM for 2 min at 95 °C, as reported by the manufacturer, and stored at −80 °C until next mass spectrometry analysis. Intracellular fractions of untreated BT474 cells, were used as negative control. Protein amounts were determined with the Pierce BCA kit (Perbio Science, Bonn, Germany).

MS analyses were performed using a ESI Ion Trap HCT ETD II HC Ultra PTM discovery system Bruker mass spectrometer coupled with an HPLC System Alliance e2695 separation module fitted out with a 2998 PDA detector (Waters, Milan, Italy). Chromatographic separations of samples (50 μL injected) were performed on an Alliance HT WATERS 2795 system, equipped with a PDA WATERS detector 2996 using a C18 Waters xBridge (3 mm, 4.6 × 5.0 mm) column. The optimized mobile phase consisted of water containing 0.1% TFA (solvent A) and CH_3_CN containing 0.1% TFA (solvent B). The elution was carried out by using a linear gradient from 1% to 70% of solvent B over 20 min, from 70% to 95% of solvent B over 1 min, following an isocratic step at 95% B for 5 min, and a re-equilibration step at 2% B for 4 min was always performed before injections. The total analysis time was thereby kept at 30 minutes.

The ESI source operating parameters were set as follows: positive ionization mode; capillary voltage 3.5 kV; source temperature 150 °C; desolvation temperature 600 °C; desolvation nitrogen gas flow rate 1000 L/h; cone gas 50 L/h; sampling cone voltage 40 V. The acquisitions were made in MRM/SRM (multiple reaction monitoring/selected reaction monitoring)^48^ mode with a dwell time of 63 ms and a collision energy of 25 V. For the SRM mode the following transitions was monitored: biotinylated-A9 at m/z 615.0–477.2.

### Computational Methods

The 3D structure of the complex between HER2-DIVMP and ^111^In-DTPA-A9 was modelled. The atomic coordinates of ^111^In-DTPA were retrieved from CSD (The Cambridge Structural Database)^53^, code XIKVES^54^, whereas a representative conformation of the complex A9/HER2-DIVMP was extracted from the most populated cluster of structures obtained by our previous Molecular Dynamics study^52^. Subsequently, the model complex between HER2-DIVMP and ^111^In-DTPA-A9 was built using the Builder module of Insight II package and was subjected to energy minimization by 1000 steps of CG. MOLMOL package was used for visual inspection and images production^62^.

### Radiolabeling chemistry and HPLC analysis

Before labelling, freeze-dried DTPA-A9 or DTPA-SA9 was reconstituted in 0.2 M ammonium acetate, pH 5.5, to a concentration of 2 mg/ml. Aliquots containing 30 µg of conjugate were stored frozen at −20 °C. For labelling, an aliquot of a conjugate was mixed with a predetermined amount of ^111^In-chloride solution. The reaction mixtures were incubated at room temperature for 60 min. After 60 min incubation, the radioconjugate radiochemical yield was measured using HPLC. For a blank experiment, ^111^In-chloride was incubated with 0.2 M ammonium acetate, pH 5.5, without DTPA-A9 for 60 min, and the mixture was analyzed using radio-HPLC.

Analytic LC was performed using an HPLC system from Beckman consisting of a 126 pump, a 166 UV detector (set at wavelength of 220 nm), and a radiation detector coupled in series. The column used was a VydacRP C18 protein column (3 × 150 mm). The applied gradient elution had the following parameters, A: 0.1% TFA in H_2_O; B: 0.1% TFA in CH_3_CN, 1 mL/min flow rate using a linear gradient starting from 5% to 80% B in 20 min. Data acquisition and handling were performed using the Beckman System Gold or Hitachi Chromaster HPLC systems.

For stability test, murine blood plasma was prepared from whole blood by centrifuging for 10 min at 2000 g (4 °C). A sample of freshly labeled ^111^In-DTPA-A9 (10 µL) was mixed with the plasma (200 µL). Samples were incubated at 37 °C. At 15 or 60 min after incubation start, pre-chilled acetonitrile (200 µL) was added to the sample to precipitate blood proteins, the sample was centrifuged for 10 min (10000 g, 4 °C). The supernatant was collected, diluted with water (1:1) and analyzed with radio-HPLC as described above. Two experiments per data point were performed to check reproducibility.

### Measurements of affinity of ^111^In-DTPA-A9 peptide binding to living HER2-expressing cells using LigandTracer Yellow

For cell studies, the HER2-expressing breast carcinoma cell line BT474 (from ATCC) was used. The cell line was cultured in RPMI medium (Flow Irvine, UK) supplemented with 10% fetal calf serum (Sigma, USA), 2 mM L-glutamine and PEST (penicillin 100 IU/mL and 100 μg/mL streptomycin), all from Biokrom Kg, Germany.

Cells from the human breast carcinoma cell line BT474 were seeded on a local area of a cell culture dish (NunclonTM, Size 100620, NUNC A/S, Roskilde, Denmark), as described by Björke and Andersson^63^. The binding of ^111^In-DTPA-A9 to living cells was monitored in real-time at room temperature using LigandTracer Yellow, using method established by Björke and Andersson^63^. In brief, the cell culture dish was placed on the sloping and rotating cell dish holder in LigandTracer Yellow, followed by a continuous monitoring of the radioactivity level along the rim of the dish. If the labelled peptide binds to the cells, an elevated signal will be recorded when the cells pass by the detector. This makes it possible to accurately detect binding events both during incubation and after wash. In order to cover the concentration span needed for proper affinity estimation entirely, three increasing concentrations 4, 12, and 36 nM (selected based on the fluorescence spectroscopy results). Each concentration was incubated long enough to approach steady state. Thereafter, the ligand solution was replaced with fresh medium and the dissociation rate was followed overnight. The data were corrected for decay of indium-111. Binding and dissociation curves were analyzed by InteractionMap software as described by Björkelund *et al*. to calculate affinity of ^111^In-DTPA-A9 binding to living HER2-expressing cells^56^.

Additionally, specificity of ^111^In-DTPA-A9 binding to HER2 expressing cells was evaluated using pre-saturation of receptors by trastuzumab and by using ^111^In-labeled scrambled DTPA-A9 (^111^In- DTPA-S-A9). The protocol for ^111^In- DTPA-S-A9 was exactly as for ^111^In-DTPA-A9. In the case of pre-saturation experiment, trastuzumab was added to cell dishes to the concentration of 400 nM and cells were incubated for 60 min immediately before affinity measurements, The medium was withdrawn and new medium was added. Thereafter, binding affinity of ^111^In-DTPA-A9 was measured using exactly the same protocol as described above. Two experiments per data point were performed to check reproducibility.

### *In vivo* studies

The animal experiment was planned and performed in accordance with Swedish national legislation on laboratory animals’ protection and was approved by the Ethics Committee for Animal Research in Uppsala, Sweden.

Four BALB/C nu/nu mice were used for the biodistribution studies. At the time of experiment, the average animal weight was 20.5 ± 0.9 g.

Animals were injected intravenously (tail vein) with 3 µg (30 kBq) of ^111^In-DTPA-A9 in 100 µL PBS. Since DTPA-A9 peptide resulted stable to serum proteolytic degradation in a range of 90 minutes (for details see Supplementary Information Fig. S6), at 1 hour after injection, a mixture of Ketalar-Rompun (20 µL of solution per gram body weight; Ketalar: 10 mg/mL; Rompun: 1 mg/mL) was injected intraperitonealy, and the mice were euthanized by a heart puncture using a syringe, pre-washed with diluted heparin (5000 IE/mL). Blood as well as brain, stomach, pancreas, lung, liver, spleen, kidneys, samples of muscle and bone, gastrointestinal tract (with its content) and remaining carcass were collected in pre-weighed plastic vials. Organs and tissue samples were weighed and measured for radioactivity using an automatic gamma counter. The tissue uptake values were calculated as per cent injected activity per gram of tissue (% ID/g) and per whole sample (% ID/sample). The gastrointestinal tract and the carcass uptakes were calculated only as % ID per whole sample. The radioactivity in the gastrointestinal tract with its content was used as a measure of hepatobiliary excretion.

Additionally, *in vivo* stability to proteolytic enzymes anchored to the vasculature walls was assessed using to method described earlier^61^. Briefly, 10 MBq of ^111^In-DTPA-A9 were injected in NMRI mice via tail vein. At 5 or 15 min, animals were sacrificed, blood was withdrawn and transferred to a EDTA-containing vial. Each sample was centrifuged for 10 min (2000 g, 4 °C), and a supernatant was collected. A pre-chilled acetonitrile (200 µL) was added to the supernatant to precipitate blood proteins, the sample was centrifuged for 10 min (10000 g, 4 °C). The supernatant was collected, diluted with water (1:1) and analyzed with radio-HPLC as described above. Two experiments per data point were performed to check reproducibility^63^.

## Electronic supplementary material

Supplementary Information
