# Supplementary material for: Evaluation of HER2-specific peptide ligand for its employment as radiolabeled imaging probe
Source: Sci Rep. 2018 Feb 14;8:2998. doi: 10.1038/s41598-018-21283-3 (PMC5812989; doi:10.1038/s41598-018-21283-3)
Supplement: Supplementary file 1 — Supplementary Information [file 41598_2018_21283_MOESM1_ESM.doc]

**Supplementary Information**

**Evaluation of HER2-specific peptide ligand for its employment as radiolabeled imaging probe**

Hadis Honarvar^1^, Enrica Calce^2,#^, Nunzianna Doti^2,#^, Emma Langella^2^, Anna Orlova^3^, Jos Buijs^3^, Valentina D’Amato^4^, Roberto Bianco^4^, Michele Saviano^5^, Vladimir Tolmachev^6^, Stefania De Luca^2,*^

^1^Department of Surgical Sciences, Radiology, Uppsala University Hospital, Uppsala, Sweden

^2^Institute of Biostructures and Bioimaging, National Research Council, Naples, Italy

^3^Division of Molecular Imaging, Department of Medicinal Chemistry, Uppsala University, Uppsala, Sweden

^4^Department of Clinical Medicine and Surgery, University of Naples "Federico II", Naples, Italy

^5^Institute of Crystallography, National Research Council, Bari, Italy

^6^Department of Immunology, Genetics and Pathology, Uppsala University, Uppsala, Sweden

^#^Authors contributed equally

*E-mail: stefania.deluca@cnr.it

**Figure S1:** LC-MS characterization of the biotinylated-A9 synthetic peptide. (**A**) HPLC profile of the biotinylated-A9 synthetic peptide using a C18 Waters xBridge (3μm, 4.6 x 50 mm) column, with a linear gradient from 5-70 of 0.05% TFA in CH_3_CN (solvent B) over 20 min at a flow rate of 0.2 mL/min. Solvent A was 0.05% TFA in H_2_O. Green and red lines represent the chromatogram at 214 and 280 nm, respectively. The blue line represent the TIC (total ion current) chromatogram. (**B**) LC-MS profile of the biotinylated-A9 peptide. The t_R_ value was 20.5 nm for the target peptide at m/z: 615.1 [M+2H]^2+^. (**C**) In-source-fragmentation mass spectra corresponding to the MS peak at m/z: 615.1. (**D**) Extracted ion chromatogram of LC/MS/MRM (transition 615.0 -- 477.2) of chromatographic peak at t_R_ 20.5.

**Figure S2:** LC-MS analysis of purification steps using the cellular extract obtained from BT474 treated with the biotinylated-A9 synthetic peptide, using a C18 Waters xBridge (3μm, 4.6 x 50 mm) column, with a linear gradient from 5-70 of 0.05% TFA in CH_3_CN (solvent B) over 20 min at a flow rate of 0.2 mL/min. Solvent A was 0.05% TFA in H_2_O. (**A**) LC-MS profile (time range between 19.0 – 22.0) of the cellular lysate not retained on streptavidin-coated resin, (**B**) of washing and (**C**) elution step. The asterisk (*) indicates the MS peak of the target peptide (m/z: 615.1 [M+2H]^2+^). (**D**) In-source-fragmentation mass spectra corresponding to the MS peak at m/z: 615.1.

**Figure S3:** LC-MS analysis of purification steps using the cellular extract obtained from untreated BT474, used as negative control, using a C18 Waters xBridge (3μm, 4.6 x 50 mm) column, with a linear gradient from 5-70 of 0.05% TFA in CH_3_CN (solvent B) over 20 min at a flow rate of 0.2 mL/min. Solvent A was 0.05% TFA in H_2_O. **(A)** LC-MS profile (time range between 19.0 – 22.0) of the cellular lysate not retained on streptavidin-coated resin, (**B**) of washing and (**C**) elution step.

**
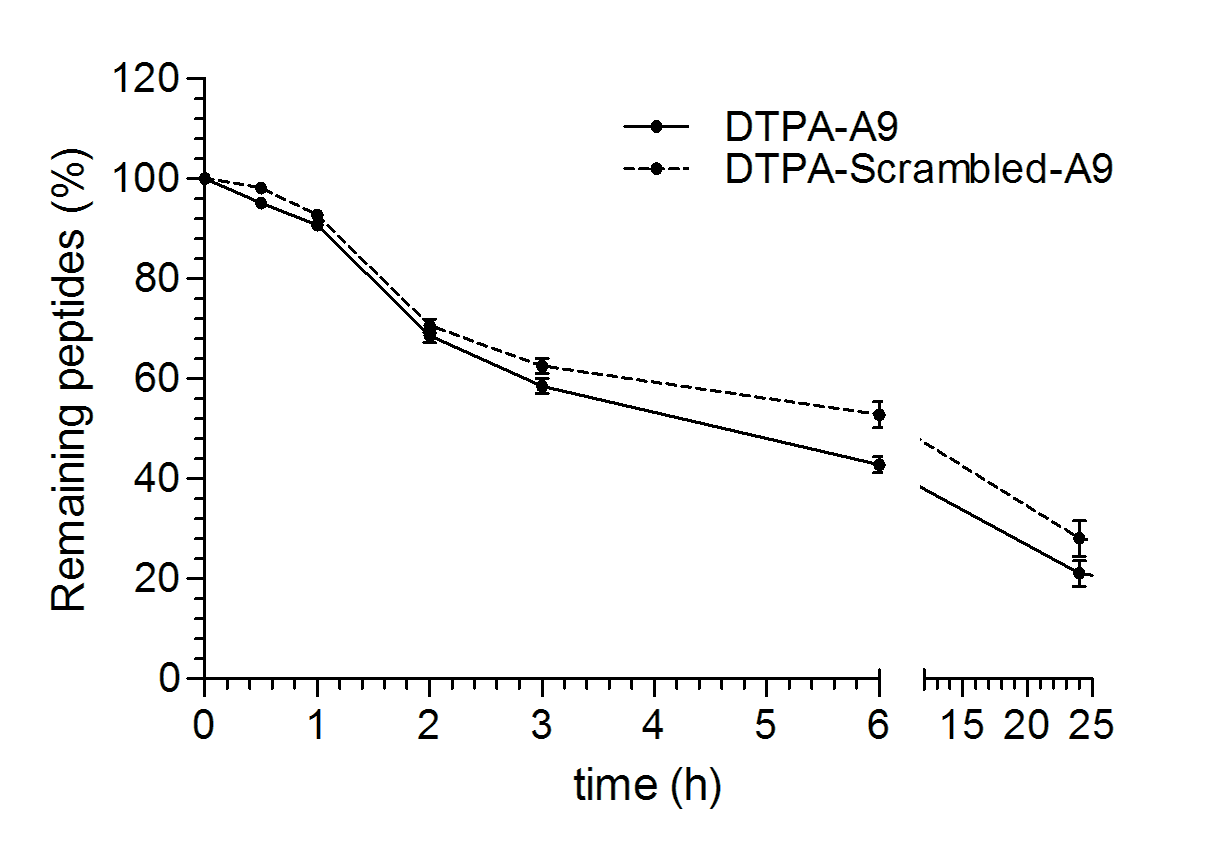
**

**Figure S4:** *In vitro* Peptide Stability in Serum. A9 modified peptides at concentration of 0.5 mg/mL were dissolved in RPMI containing 10% of FBS (fetal bovine serum) and incubated at 37 °C. Samples (50 μL) were taken regularly (*t* = 0, 0.5, 1, 2, 3, 6, and 24 h) and mixed with 100 μL of CH_3_CH_2_OH (96%), for precipitation of serum proteins. The cloudy reaction sample is cooled (4 °C) for 15 min and then spin at 16,000 rpm for 2 min, to pellet the precipitated serum proteins^1^. The chromatographic analysis of samples (30 μL injected for each injection) were performed on an Alliance HT WATERS 2795 system, equipped with a PDA WATERS detector 2996 using a C18 Waters xBridge (3 μm, 4.6x5.0 mm) column using a C18 Waters xBridge (3 μm, 4.6 x 50 mm) column, with a linear gradient from 5% to 70% of 0.05% TFA in CH_3_CN (Solvent B) over 20 min at a flow rate of 0.2 mL/min. The percentage of peptide serum stability was calculated by integration of HPLC chromatographic peak area of intact peptide revealed at 210 nm. Results are mean of three independent experiments performed in duplicate.

**A**

**
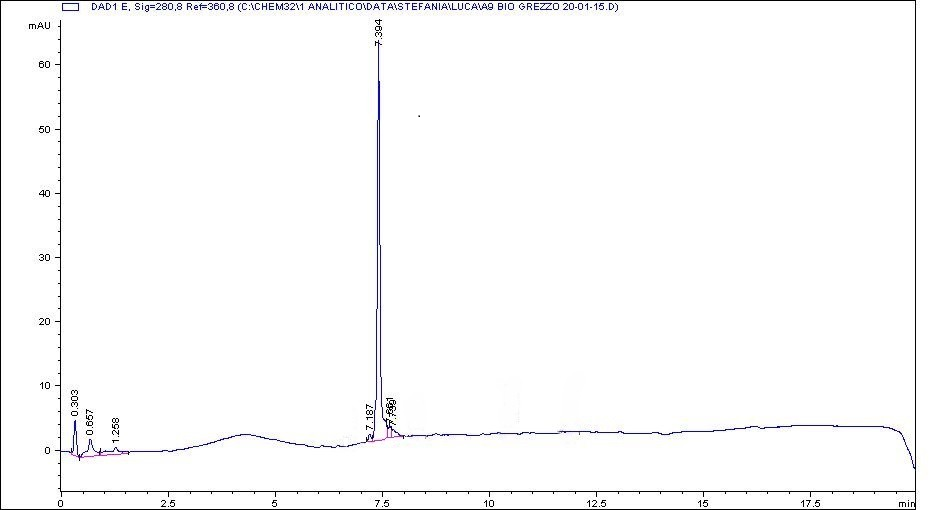
**

**B**

5

x10

0

0,1

0,2

0,3

0,4

0,5

0,6

0,7

0,8

0,9

1

1,1

1,2

1,3

1,4

1,5

1,6

1,7

1,8

+ESI Scan (12,098; 12,513 min, 2 Scans) Frag=200,0V Biotinylated-A9.d

614,8359

1228,6641

159,1037

1157,6229

1025,5423

502,3499

954,5030

Counts vs. Mass-to-Charge (m/z)

150

200

250

300

350

400

450

500

550

600

650

700

750

800

850

900

950

1000

1050

1100

1150

1200

1250

1300

1350

1400

1450

1500

1550

1600

1650

1700

1750

1800

1850

1900

1950

**Figure S5:** HPLC profile and MS spectrum of Biotinylated-A9. (A) HPLC analysis was carried out using a C18 column, 250*4.6 mm ID (Phenomenex, Torrance, CA, USA) at a flow rate of 1.0 mL min^-1^. The t_R_ (retention time) value of desired product was 7.4 min. (B) MS analysis showed the expected mass for Biotinylated-A9 at m/z: 1228.664 [M+H]^+^ and m/z: 614.836 [M+2H]^2+^.

**A**

**
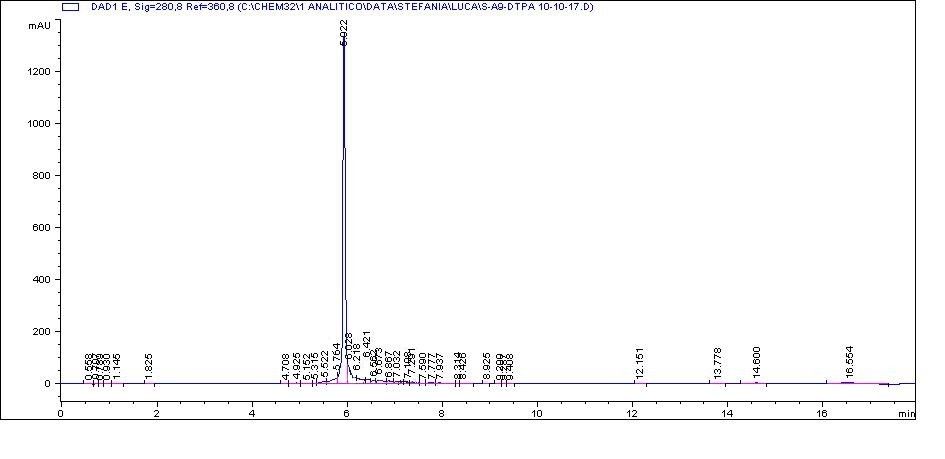
**

**B**

6

x10

0

0,1

0,2

0,3

0,4

0,5

0,6

0,7

0,8

0,9

1

1,1

1,2

1,3

1,4

1,5

1,6

+ESI Scan (10,882-11,048 min, 11 Scans) Frag=200,0V DTPA-Scrambled-A9.d

1377,7246

689,3757

1430,6368

623,8250

538,7665

Counts vs. Mass-to-Charge (m/z)

150

200

250

300

350

400

450

500

550

600

650

700

750

800

850

900

950

1000

1050

1100

1150

1200

1250

1300

1350

1400

1450

1500

1550

1600

1650

1700

1750

1800

1850

1900

1950

**Figure S6:** HPLC profile and MS spectrum of DTPA-SA9. (A) HPLC analysis was carried out using a C18 column, 250*4.6 mm ID (Phenomenex, Torrance, CA, USA) at a flow rate of 1.0 mL min^-1^. The t_R_ (retention time) value of desired product was 5.9 min. (B) MS analysis showed the expected mass for DTPA-SA9 at m/z: 1377.724 [M+H]^+^ and m/z: 689.375 [M+2H]^2+^.

**References**

1. Jenssen, H., Aspmo, S.I. Serum stability of peptides. *Methods Mol Biol.* **494**, 177-186 (2008).
